# Wellness-enhancing effects of the canine growth hormone releasing hormone therapy mediated by plasmid and electroporation in healthy old dogs

**DOI:** 10.3389/fvets.2025.1609405

**Published:** 2025-09-15

**Authors:** Min-Ok Ryu, Sung-moon Kim, J. Joseph Kim, Hyun Namkung, Ho Kyoung Jung, Hee Jin Nam, Charles C. Reed, Ga-Hyun Lim, Eun Jin Kim, Hwa-Young Youn

**Affiliations:** ^1^Laboratory of Internal Medicine, College of Veterinary Medicine and Research Institute for Veterinary Science, Seoul National University, Seoul, Republic of Korea; ^2^Global R&D Center, Plumbline Life Sciences, Inc., Seoul, Republic of Korea; ^3^AGENTA Therapeutics, Blue Bell, PA, United States; ^4^Research and Development Center, Hongcheon CTCVAC Co., Ltd., Hongcheon, Republic of Korea; ^5^Independent Consultant, Soudertorn, PA, United States

**Keywords:** growth hormone-releasing hormone, immune-enhancing, immunosenescence, aging, dog

## Abstract

Aging leads to increased disease susceptibility and weakened immunity, a condition known as immunosenescence. The growth hormone-releasing hormone (GHRH)/growth hormone (GH)/insulin-like growth factor 1 (IGF-1) axis plays a key role in both somatic growth and immune modulation. This study evaluated the clinical and immunological effects of a canine GHRH-encoding plasmid delivered by electroporation in 30 healthy senior dogs (aged 10–16 years). Dogs received a single intramuscular injection and were monitored over 180 days. Significant improvements were observed in clinical scores, with 90% of dogs showing increased well-being based on owner-assessed measures including appetite, activity, and exercise tolerance. Limb thickness, used as a surrogate for muscle mass, significantly increased in both hindlimbs by day 180. While mean serum IGF-1 concentrations did not change overall, post-hoc stratification revealed that dogs with low baseline IGF-1 (<90 ng/mL) showed substantial increases, whereas those with high baseline levels tended to decrease. This bidirectional modulation suggests feedback-sensitive regulation of the GHRH-GH-IGF-1 axis. Flow cytometry demonstrated increases in total CD3+ T cells, as well as naïve CD4+ and CD8+ T cell subsets, indicating a potential delay in immunosenescence. The therapy was well-tolerated, with no serious adverse effects reported; hematologic abnormalities and gastrointestinal symptoms were transient and resolved without intervention. These findings suggest that GHRH-encoding plasmid therapy may improve clinical condition and modulate immune function in aging dogs, warranting further investigation into its long-term efficacy and potential applications.

## 1 Introduction

Aging increases vulnerability to diseases such as cancer, infections, inflammatory and autoimmune disorders in both humans (1) and animals (2, 3). Immunosenescence, or immune aging, is the decline of immune system functions associated with aging, and represents a growing area of importance for medical research. Immunosenescence is associated with thymic involution resulting in loss of immune function resulting in a decline in naïve immune cell production from the bone marrow and thymus (3–5). Human aging is associated with significant immunological alterations, such as reduced CD3+ and CD4+ T-cell counts, associated with an increase in CD8+ T cells, a lowered CD4: CD8 ratio, and a decline in naïve T-cell populations (6). Research indicates that dogs exhibit immunosenescence patterns akin to humans, including elevated cytokine levels, reduced CD3+ and CD4+ T cells, a rise in CD8+ T cells, a lower CD4: CD8 ratio, a decline in naïve CD4+ and CD8+ T cells (CD45RA+ CD62L+), and diminished proliferation of CD4+ and CD8+ T cells (2, 3, 7–9).

The GHRH/GH/IGF-1 axis is intricately linked with the immune system, a relationship often described as the immune-endocrine loop or immunoendocrine interaction (10, 11). Extensive research has demonstrated that GHRH, GH, and IGF-1 influence various immune functions, including innate cell activity, B lymphopoiesis, B cell immunoglobulin synthesis, thymopoiesis, and thymic output (10, 11). In addition to supporting B cell development, this axis plays a significant role in T cell homeostasis. GH and IGF-1 have been shown to enhance thymic output and maintain naïve T cell populations, particularly in older individuals where thymic involution impairs T cell renewal (12, 13). Recombinant GH therapy has been associated with increased counts of CD4^+^ and CD8^+^ naïve T cells in elderly and immunocompromised patients, suggesting a potential for reversing aspects of immunosenescence (12, 14). These findings support the rationale for exploring whether GHRH plasmid therapy can modulate T cell subsets in aging dogs.

The aging process in both humans and animals is accompanied by functional declines in the GHRH/GH/IGF-1 axis, leading to reduced GH secretion and subsequent decreases in IGF-1 levels (12, 13, 15). Therapies targeting this axis—such as GHRH or GH administration—have been reported to bolster immune function (14, 16–18), drawing increasing interest as potential interventions to counteract immunosenescence and promote healthy aging.

In this veterinary trial, we investigated the treatment effects of canine GHRH encoding plasmid therapy delivered by electroporation on healthy older dogs. Specifically, we assessed the clinical indicators of aging and wellness as well as the changes in naïve T cell populations and IGF-1 concentrations. By administering GHRH encoding plasmid, we also sought to explore its potential to modulate immunosenescence-related changes and offer insights into the interplay between immunosenescence and the GHRH/GH/IGF-1 axis in aging canines.

## 2 Materials and methods

### 2.1 Study design

The clinical trial was approved by the Institutional Animal Care and Use Committee (IACUC approval #: DWP-IACUC-001) and was conducted in collaboration with veterinarians from four veterinary clinics. Each blood sample was delivered to the central lab and FACS analysis center for each evaluation item as requested sample form and analyzed according to each SOP, respectively.

**Figure 1:**
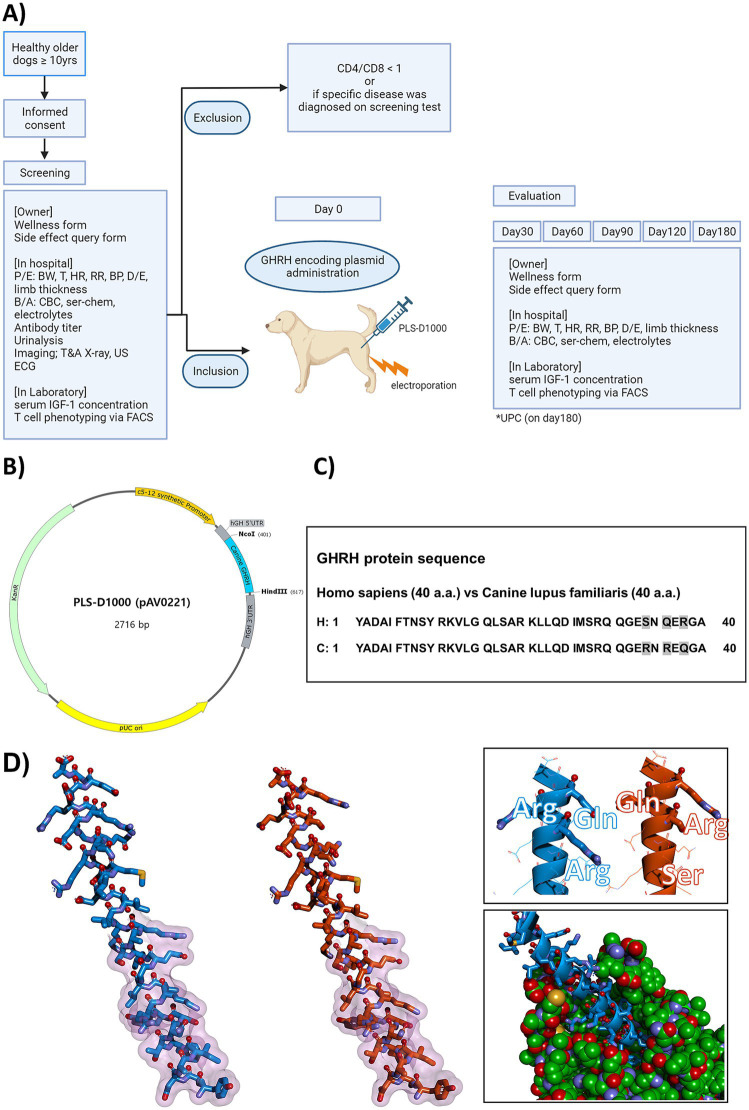
Comprehensive Illustration of Canine GHRH Study: Clinical Trial Scheme, Plasmid Design, Homology Comparison, and Molecular Models. **(A)** The scheme of the clinical trial in this study. P/E, physical examination; BW, body weight; T, temperature; HR, heart rate; RR, respiratory rate; BP, blood pressure; D/E, dermal examination; B/A, blood analysis; CBC, complete blood counts; ser-chem, serum chemistry; T&A, thoracic and abdominal; US, ultrasound; ECG, electrocardiography; IGF-1, insulin-like growth factor 1; FACS, Fluorescence-activated cell sorting; GHRH, growth hormone releasing hormone; UPC, urine protein creatinine ratio. **(B)** Plasmid DNA map and DNA sequence of canine GHRH encoding plasmid (PLS-D1000, pAV0221). Using the backbone of the pVAX1tm vector (Invitrogen, Cat. No. V260-20), muscle-specific promoter (c5-12 synthetic promoter, 13–335), hGH 5’UTR (348–402), canine GHRH (403–615), hGH 3’UTR (626–817) was designed and inserted. **(C)** Comparison of homology between human GHRH and canine GHRH. Human GHRH protein and canine GHRH protein each have 40 amino acids. Human and canine GHRH proteins have an identity of 92.5% (37/40) and a similarity of 97.5% (39/40). C, *Canis lupus familiaris*; H, *Homo sapiens*. **(D)** Molecular model of the mature canine GHRH peptide from pAV0221 (left, stick format in blue) and a model of the similar region of human GHRH (center, stick format in orange) for comparison purposes. Regions of the peptide that putatively interact with receptor are indicated by a transparent fuchsia surface. The nonidentical residues between the two peptides are shown in a different perspective as sticks in the top right inset, and a model of the canine peptide (blue) docked into a canine GHRH receptor (green cpk format) is shown in the inset bottom right.

Dogs qualified for inclusion if they were over 10 years of age, completely vaccinated, free from particular diseases, and showed reduced appetite or activity levels. Following informed consent from owners, a thorough assessment was performed, including physical and ocular exams, thoracic and abdominal radiographs, abdominal ultrasound, electrocardiography, complete blood counts (CBC), serum chemistry, electrolytes, and urinalysis. Blood sampling was performed in the morning after an overnight fast, and tests were conducted at approximately the same time of day at each hospital visit to minimize temporal variation. Flow cytometry was used to assess CD4 and CD8 levels in EDTA-anticoagulated whole blood. Dogs were excluded if they presented abnormal checkup results or a CD4/CD8 ratio below 1 in flow cytometry. After evaluation on day 0, each dog was administered a single injection of a plasmid optimized for expressing the canine GHRH gene. Post treatment all dogs were observed at the animal hospital on days 0, 30, 60, 90, 120, and 180. Assessments at each time point included CBC, serum chemistry, weight, body temperature, heart and respiratory rates, body condition score, capillary refill time, blood pressure, limb thickness, serum IGF-1, and CD3/CD4/CD8 blood levels. The Urine Protein Creatinine (UPC) ratio was assessed both before treatment on day 0 and after treatment on day 180. At each visit, the veterinarian interviewed the owner, recorded health conditions and adverse events, and completed a wellness form. The wellness form was created using insights from a study on age-related physical and functional changes in senior dogs, (19) as well as the Ohio State University Veterinary Medical Center Honoring the Bond Program Quality of Life Checklist (20) and HHHHHMM scale (21). The wellness form included assessments of activity level, exercise tolerance, mentation (attitude, alertness), play, interaction, appetite, thirst, frequency of relaxed postures, hair brightness, skin dryness, skin odor, sleep patterns, and overall quality of life. Veterinarians rated their dog’s condition on a scale from very good (5) to very poor (1) (as shown in Table 1). The clinical score, representing the total score, was computed for subsequent analysis. Apart from the clinical score, an owner questionnaire evaluated symptom changes since the last assessment, concentrating on appetite, activity, and mobility. Scores were assigned as follows: 1 indicates a significant decrease, 2 a decrease, 3 no change, 4 an increase, and 5 a significant increase. The adverse events form included counts of nausea, diarrhea, appetite loss, exercise intolerance, and the presence of urticaria, facial edema, and dyspnea. Veterinarians monitored each dog from immediately after the injection until full recovery from anesthesia, checking for any abnormalities in vital parameters and for local adverse reactions at the injection site. Follow-up evaluations were conducted on days 30, 60, 90, 120, and 180, during which veterinarians assessed the injection site for adverse reactions (including pain, erythema, granulomas, and ulcers), monitored vital signs, and reviewed hematologic test results. At each follow-up visit, owners were also surveyed regarding any observed adverse events at home. Prior to the clinical trial, an extensive owner education program informed participants about possible adverse events. The possibility of terminating the clinical trial was highlighted should severe adverse events arise. This clinical trial’s scheme is summarized in Figure 1A.

**Table 1:** The wellness form for clinical scoring of dogs.

Questionnaire	day 0	day 30	day 60	day 90	day 120	day 180
How was your dog’s overall health in the last period?						
How has your dog been feeling over the past period?						
How was your dog’s attitude over the last period?						
Did your dog still do his favorite things like walk and play during the last period?						
How often did your dog show a relaxed posture in the last period?						
What was your dog’s activity in the last period?						
How much did your dog try to play with itself in the past?						
How often has your dog exercised in the past?						
Has your dog been tiring easily in the past?						
How well did your dog eat his food in the last period?						
How well drink your dog eat water in the past?						
Has your dog’s coat gotten shinier over the past period?						
Has your dog’s skin become less flaky over the past period?						
Has your dog’s skin odor decreased further over the past period?						
How much did your dog want to be with your family in the past?						
What has your dog’s sleep activity been like in the past?						
Total						

Very bad: 1, bad: 2, acceptable: 3, good: 4, very good: 5.

### 2.2 Design of canine GHRH plasmid

A model of the processed canine peptide GHRH model was prepared with Discovery Studio 2021 (Biovia, San Diego) and DeepView v4.1 (22). The PLS-D1000 (pAV0221) plasmid was constructed using a self-constructed vector containing the muscle-specific promoter SPc5-12, human GH 5′ untranslated region, and human GH 3′ untranslated region based on the pVAX1^tm^ vector (Invitrogen, Cat. No. V260-20) as a backbone, and the canine GHRH expression gene was inserted using the NcoI/HindIII sites (Figure 1B). The human processed GHRH peptide model was derived from the AlphaFold Protein Structure Database entry AF-P01286-F1 (Figures 1C,D). Residues putatively interacting with GHRHR were derived from homology with structures of human GHRH bound to receptors [7v9m.pdb (23) and 7cz5.pdb (24)]. Predicted bound structure of canine GHRHR (Uniport acc. A0A8C0MLJ3) with canine GHRH was generated with Discovery Studio 2021 and DeepView v4.1 using 7v9m.pdb as a template.

### 2.3 Administration of canine GHRH plasmid

The plasmid encoding synthetic canine GHRH is supplemented with 1% (w/w) poly-L-glutamic acid sodium salt and diluted to a concentration of 1 mg/mL using sterile water. Dogs were anesthetized using a mix of butorphanol (0.1–0.4 mg/kg) and midazolam (0.1–0.3 mg/kg), followed by a 1 mL subcutaneous injection of 2% lidocaine at the site. The 1 mg/mL plasmid was injected into the dog’s hind quadriceps femoris muscle, and the administration was completed immediately using an electroporation device. A previous study has reported that when plasmids are introduced into muscle cells via electroporation, the gene expression effect may persist for approximately 6 months (25). Electroporation at the muscle injection site utilized the CELLECTRA® 5P adaptive *in vivo* EP system and the 5P IM applicator from INOVIO Pharmaceuticals Inc., Plymouth Meeting, PA. USA at 0.5 Amps, 3 pulses of 52 milliseconds each, 1 s between pulses (26). After injection, the animals were observed by a veterinarian until they recovered from anesthesia. Afterwards, the owners were educated to observe adverse effects, and the injection site was checked for any abnormalities at each time point by veterinarians.

### 2.4 Measurement of IGF-1 concentrations

Blood samples were collected from the canines at baseline, and on days 30, 60, 90, 120, and 180. Blood samples were processed to isolate serum and were stored at −80 °C for subsequent analysis. Serum IGF-1 levels were measured by GCCL Co., Ltd. using immunoassay methods with the Cobas 8000® e801 module from Roche Diagnostics, Basel, Switzerland, adhering to established protocols.

### 2.5 Flow cytometry

The blood collected in EDTA tubes was sent to the FACS analysis center (Global R&D Center of Plumbline Life Science, Inc.) within an hour and immediately performed the experiment by the same operator using the same FACS instrument to maintain data consistency. Each used antibodies were prepared with the same lot number respectively, preventing the data variation. Additionally, the optimal usage amount for each antibody used was determined through prior testing.

Blood sample was centrifuged at 500 g for 10 min. Subsequently, RBC lysis buffer (R2035, BIOSESANG, Korea) was applied to the resulting pellet and incubated at room temperature for 10 min. Cells were washed with PBS and transferred to a FACS tube at a concentration of 1 × 106 cells per tube. Cells were washed twice with PBS containing 1% FBS and subsequently stained with antiCD3-FITC (BIO-RAD) (27) antiCD4-PECY7 (Invitrogen) (7), antiCD8-PB (BIO-RAD) (7), antiCD62L-PE (BIO-RAD) (7, 28), antiCD45RA (BIO-RAD) (7), and antiCD45RA-APC (as shown in Table 2). The T-cell subset gating strategy commenced with the identification of CD3+ CD4+ (CD4+ T cells) and CD3+ CD8+ (CD8+ T cells) populations. Naïve (CD45RA+ CD62L+), central memory (CD45RA-CD62L+), effector memory (CD45RA − CD62L−), and terminally differentiated effector memory (CD45RA+ CD62L−) T cells were identified within CD3+ CD4+ and CD3+ CD8+ subsets based on CD45RA and CD62L expression. Fluorescence was measured using a BD CANTO II instrument and analyzed using BD FACSDiva Software v9.4.

**Table 2:** List of canine-specific and cross-reactive mAb combinations and antigen detection assays used for flow cytometric evaluation of canine peripheral blood lymphocytes.

Antigen	1st incubation mAb added	2nd incubation mAb added	Cat #	Lot #
CD3	RAT ANTI HUMAN CD3: FITC	-	MCA1477F	156,415
CD4	CD4 Monoclonal Antibody (YKIX302.9), PE-Cyanine7, eBioscience™	-	25–5,040-42	2,382,863
CD8	RAT ANTI DOG CD8: Pacific Blue®	-	MCA1039PB	155,524
CD45RA	MOUSE ANTI DOG CD45RA		MCA2036S	155,188
		Rat anti-Mouse IgG1 Secondary Antibody, APC, eBioscience™	17–4,015-80	2,272,708
CD62L	MOUSE ANTI HUMAN CD62L: RPE	-	MCA1076PE	154,349

### 2.6 Statistical analysis

Statistical evaluations were conducted using SPSS (Windows version 26, IBM Corp., Armonk, New York) and GraphPad Prism v10 (GraphPad Software, Inc., La Jolla, California). A *p*-value < 0.05 denoted statistical significance. Normality was assessed with the Shapiro–Wilk test. As some variables at certain time points did not meet the assumption of normality, the Friedman test was used to analyze changes in each parameter across time points (day 0, 30, 60, 90, 120, and 180). *Post hoc* comparisons with day 0 were performed using Dunn’s multiple comparison test. For CD4+ naïve T cell analysis, however, an uncorrected Dunn’s test was applied. Differences in IGF-1 levels between the Low Basal Group and High Basal Group were assessed using a t-test. The variation in response rates to GHRH based on initial IGF-1 levels was examined with a chi-squared test. Data are presented as mean ± SEM, except for patient signalment variables (age and weight), which are expressed as median (range).

## 3 Results

### 3.1 Canine subject demographics

A total of 49 elderly dogs (≥10 years old) without a diagnosis of any specific disease were initially recruited for the study. Nineteen dogs were excluded based on predefined criteria, resulting in 30 dogs being enrolled (Figure 1A). The exclusion criteria comprised a confirmed diagnosis of autoimmune or other specific diseases, participation in another clinical trial within the preceding 6 months, a CD4-to-CD8 ratio ≥1, and inability to participate for a period exceeding 4 months. Table 3 presents the demographic information of the canine subjects. The study population included 13 castrated males, 10 spayed females, 6 intact females, and 1 intact male. The median age of the population was 11 years (range: 10–16), with a median weight of 5 kg (range: 3.2–41.0). The most common breeds for the study were Maltese (*n* = 6), Poodle (*n* = 6), and Pomeranian (*n* = 4).

**Table 3:** Signalments of enrolled dogs.

Dog#	Age (years)	Sex	Weight (kg)	Breed
1	16	CM	4.8	Maltese
2	15	SF	4.8	Maltese
3	15	SF	5	Shih-tzu
4	10	SF	3.2	Poodle
5	11	SF	5.2	Poodle
6	15	SF	4.2	Shih-tzu
7	10	IF	8.3	Mixed
8	11	IF	3.7	Pomeranian
9	10	IF	4.1	Shih-tzu
10	10	CM	41	Labrador Retriever
11	10	SF	4.3	Maltese
12	12	CM	5.3	Pompitz
13	12	CM	5.45	Pomeranian
14	10	CM	7.5	Poodle
15	10	CM	5.4	Pomeranian
16	10	CM	4.7	Maltipoo
17	11	IM	10	French bulldog
18	11	IF	8.5	French bulldog
19	12	IF	5	Pomeranian
20	11	CM	6	Mixed
21	11	IF	20	Labrador Retriever
22	11	CM	4	Mixed
23	12	CM	4	Maltese
24	10	CM	4	Poodle
25	10	SF	14	Beagle
26	10	SF	3.7	Maltese
27	10	SF	5	Maltese
28	10	CM	10	Dachshund
29	11	SF	5	Poodle
30	10	CM	5.58	Poodle

CM, castrated male; IF, intact female; SF, spayed female.

### 3.2 Changes of the clinical score, appetite, activity, and exercise tolerance

Clinical scores, derived from owner-completed questionnaires on days 0, 30, 60, 90, and 180, yielded mean values of 56.17 ± 1.46, 61.00 ± 1.35, 62.87 ± 1.59, 64.67 ± 1.19, 65.03 ± 1.47, and 65.47 ± 1.10, respectively. Twenty seven of 30 treated dogs (90%) showed improvement in clinical score. A Friedman test indicated a significant change in clinical scores over time (*p* < 0.0001). Dunn’s multiple comparison test revealed significant increases in clinical scores from day 0 at day 60, 90, 120, and 180 (all *p* < 0.001).

**Figure 2:**
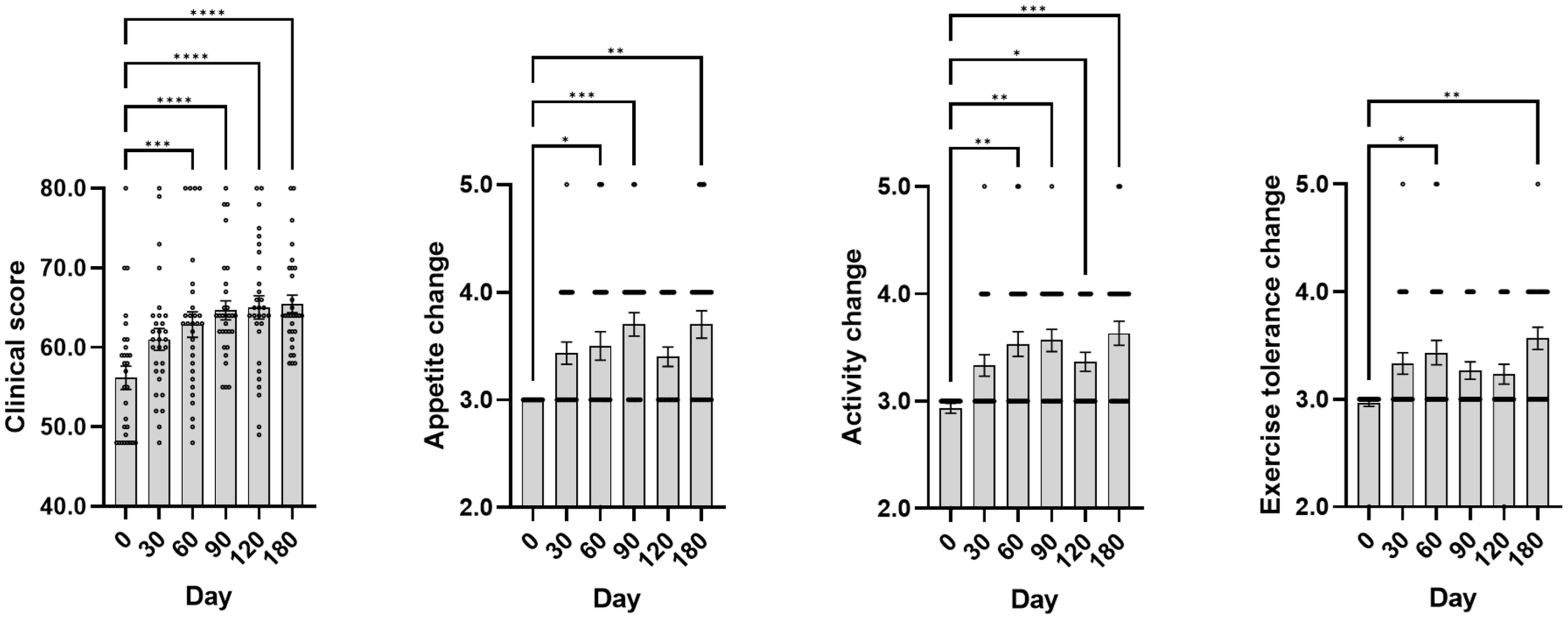
Changes in clinical, appetite, activity, and exercise tolerance scores postGHRH treatment. The day 0 values were analyzed from blood samples collected before GHRH administration, and the GHRH administration was performed on day 0. Quantitative data are presented as mean ± SEM. Normality was tested using the Shapiro–Wilk test. As some variables did not meet normality assumptions, the Friedman test was used to evaluate changes over time, and Dunn’s multiple comparison test was performed for *post hoc* comparisons with day 0. Significance markers *, **, ***, and **** correspond to *p* < 0.05, *p* < 0.01, *p* < 0.001, and *p* < 0.0001 compared to day 0, respectively. The images shown were generated using GraphPad Prism version 10.

Evaluation of appetite, activity, and exercise intolerance scores using the Friedman test revealed significant temporal differences for all three parameters (all *p* < 0.0001). Dunn’s multiple comparison test showed that appetite change scores were significantly higher at days 60, 90, and 180 (all *p* < 0.05) compared with baseline; activity change scores were significantly higher at days 60, 90, 120, and 180 (all *p* < 0.05); and exercise intolerance scores were significantly higher at days 60 and 180 (all *p* < 0.05). Scores for all parameters began to increase on day 30 and remained elevated through day 180 following injection of the GHRH-encoding plasmid. Mean clinical score, appetite change score, activity change score, and exercise intolerance change score by time point are presented in Table 4 and Figure 2.

**Table 4:** Changes in clinical score, appetite, activity, and exercise tolerance by each time points.

Day	Clinical score			Appetite change score			Activity change score			Exercise tolerance change score		
	Mean	SD SEM	*p* (vs.day0)	Mean	SD SEM	*p* (vs.day0)	Mean	SD SEM	*p* (vs.day0)	Mean	SD SEM	*p* (vs.day0)
day0	56.167	8.009 1.462	n/a	3.000	0.000 0.000	n/a	2.9333	0.25371 0.04632	n/a	2.9667	0.18257 0.03333	n/a
day30	61.000	7.409 1.353	0.125	3.433	0.568 0.104	0.072	3.3333	0.54667 0.09981	0.136	3.3333	0.54667 0.09981	0.038
day60	62.867	8.693 1.587	0.001	3.500	0.731 0.133	0.036	3.5333	0.62881 0.11480	0.004	3.4333	0.62606 0.11430	0.009
day90	64.667	6.525 1.191	0.000	3.700	0.596 0.109	0.001	3.5667	0.56832 0.10376	0.001	3.2667	0.44978 0.08212	0.067
day120	65.033	8.032 1.466	0.000	3.400	0.498 0.091	0.114	3.3667	0.49013 0.08949	0.048	3.2333	0.50401 0.09202	0.105
day180	65.467	6.033 1.101	0.000	3.700	0.702 0.128	0.002	3.6333	0.61495 0.11227	0.001	3.5667	0.56832 0.10376	0.001

SD; standard deviation, SEM; standard error of the mean. The *p*-values indicate the results of Dunn’s multiple comparison test (vs. day 0) following a Friedman test.

### 3.3 Evaluation of weight and limb thickness

**Figure 3:**
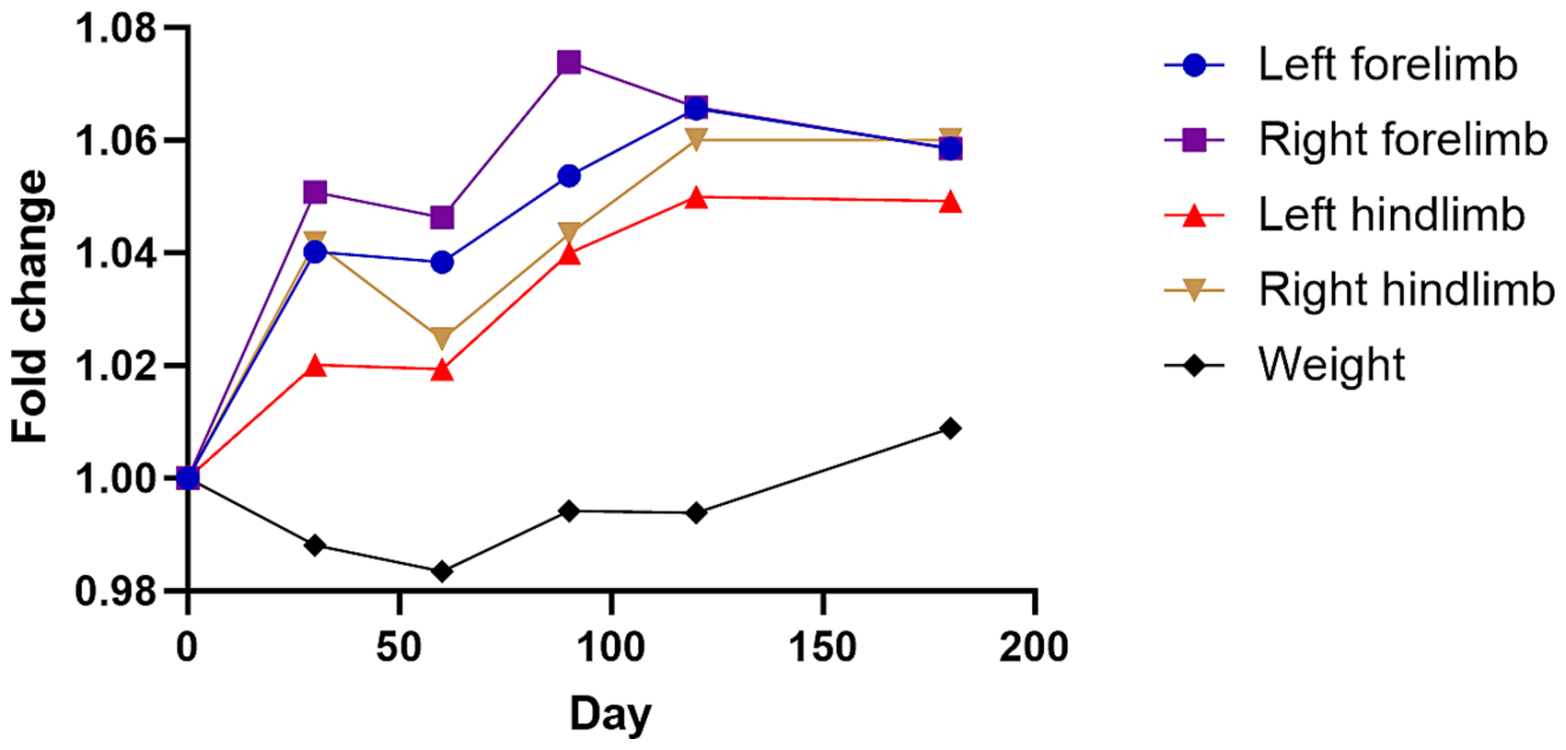
Changes in four limbs thickness and weight following GHRH therapy. Mean changes in limb thickness and weight converted to fold change over time graph. The day 0 values were analyzed from blood samples collected before GHRH administration, and the GHRH administration was performed on day 0. Normality was assessed using the Shapiro–Wilk test, and the Friedman test with Dunn’s multiple comparison test was applied to compare each time point with day 0.

Friedman test analysis revealed no significant temporal differences in body weight, left forelimb thickness, or right forelimb thickness (all *p* > 0.05) following injection of the GHRH-encoding plasmid (Table 5; Figure 3). For the hindlimbs, significant overall changes were observed in the left hindlimb (*p* = 0.0018) and right hindlimb (*p* = 0.0011). Dunn’s multiple comparison test indicated that both the left and right hindlimb thicknesses significantly increased at day 180 compared with baseline (*p* = 0.0168 and *p* = 0.0209, respectively).

**Table 5:** Body weight and limb thickness during the study.

Statistic	Left forelimb (fold change)					
	Day 0	Day 30	Day 60	Day 90	Day 120	Day 180
Mean	1.00	1.04	1.04	1.05	1.07	1.06
SD	0.000	0.106	0.143	0.225	0.254	0.250
SEM	0.000	0.019	0.026	0.041	0.046	0.045
*p* (vs. day 0)		0.891	>0.999	>0.999	>0.999	>0.999

Statistic	Right forelimb (fold change)					
	Day 0	Day 30	Day 60	Day 90	Day 120	Day 180
Mean	1.00	1.05	1.05	1.07	1.07	1.06
SD	0.000	0.113	0.149	0.227	0.257	0.249
SEM	0.000	0.021	0.027	0.041	0.047	0.045
*p* (vs. day 0)		0.949	>0.999	0.114	0.737	>0.999

Statistic	Left hindlimb (fold change)					
	Day 0	Day 30	Day 60	Day 90	Day 120	Day 180
Mean	1.00	1.02	1.02	1.04	1.05	1.05
SD	0.000	0.065	0.083	0.093	0.145	0.154
SEM	0.000	0.012	0.015	0.017	0.026	0.028
*p* (vs. day 0)		>0.999	>0.999	0.059	0.227	**0.019**

Statistic	Right hindlimb (fold change)					
	Day 0	Day 30	Day 60	Day 90	Day 120	Day 180
Mean	1.00	1.04	1.02	1.04	1.06	1.06
SD	0.000	0.090	0.099	0.125	0.153	0.167
SEM	0.000	0.16	0.018	0.023	0.028	0.030
*p* (vs. day 0)		>0.999	>0.999	0.364	0.053	**0.021**

Statistic	Weight (fold change)					
	Day 0	Day 30	Day 60	Day 90	Day 120	Day 180
Mean	1.00	0.99	0.98	0.99	0.99	1.01
SD	0.000	0.034	0.060	0.087	0.095	0.080
SEM	0.000	0.006	0.011	0.016	0.017	0.015
*p* (vs. day 0)		0.690	0.246	>0.999	>0.999	>0.999

SD; standard deviation, SEM; standard error of the mean. The *p*-values indicate the results of Dunn’s multiple comparison test (vs. day 0) following a Friedman test.

### 3.4 Serum IGF-1 concentration

**Figure 4:**
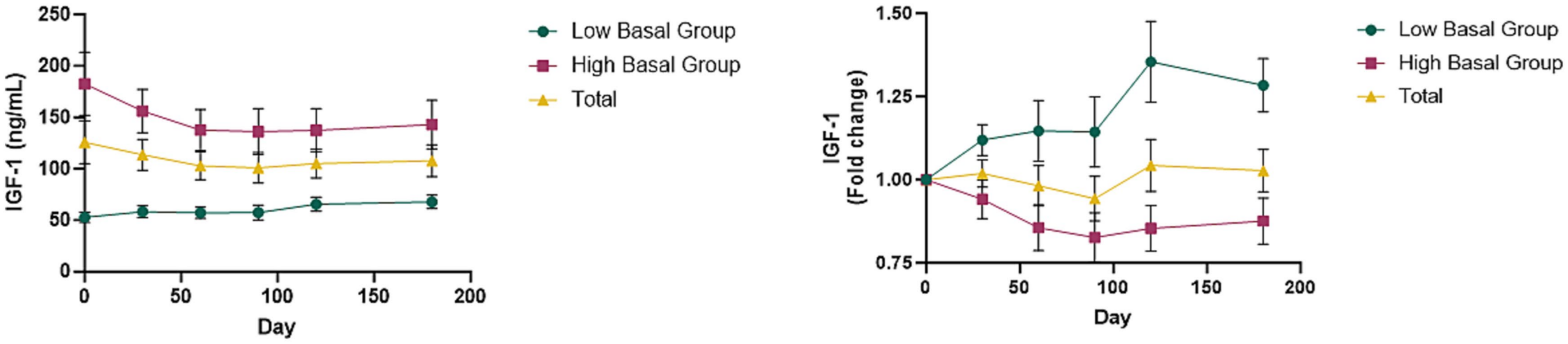
Changes of IGF-1 levels with GHRH therapy. **(A)** Mean IGF-1 levels in total dogs, Low Basal Group, and High Basal Group by time point. **(B)** Mean changes in IGF-1 levels converted to fold change over time graph. Graphs represent the mean ± SEM. The day 0 values were analyzed from blood samples collected before GHRH administration, and the GHRH administration was performed on day 0.

To investigate the effects of canine GHRH administration on the GHRH/GH/IGF-1 axis, we measured serum IGF-1 levels pre- and post-treatment. The average basal serum IGF-1 level in a group of 30 dogs was 125.81 ± 21.01 ng/mL. Mean serum IGF-1 concentration following administration of GHRH encoding plasmid did not increase over time when analyzed in bulk (Figure 4). Analyzing further, we ranked the dogs based on their basal IGF concentration at Day0. The dogs were stratified to two groups post-hoc based on a baseline IGF-1 concentration of 90 ng/mL. The first group of 13 dogs had the mean basal IGF concentration of “51.55 ± 4.70 ng/mL” (Low Basal Group) while the other 17 dogs had the mean basal IGF concentration of “182.59 ± 30.56 ng/mL” (High Basal Group). These basal IGF-1 levels between the groups were statistically significant (*t*-test; *p* = 0.001). In the Low Basal Group, 84.6% (11/13) dogs exhibited an increase in IGF-1 levels after the administration of GHRH encoding plasmid. In contrast, in the High Basal Group, 76.5% (13/17) dogs showed a decrease in IGF-1 levels following the administration of GHRH encoding plasmid.

In addition, when classified into two groups based on small dogs (less than 10 kg) in the classification by body weight of the American Veterinary Medical Association, dogs weighing less than 10 kg tended to maintain the canine baseline IGF-1 (90 ng/mL) after GHRH plasmid administration. On the other hand, in medium to large dogs (dogs weighing 10 kg or more), the baseline IGF-1 was 246.68 ng/mL, which was 2.74 times higher than the canine baseline IGF-1 (90 ng/mL), but tended to decrease after GHRH plasmid administration (up to 25% decrease) (see Supplementary Figure 1).

### 3.5 Flow cytometry results of T cells

**Figure 5:**
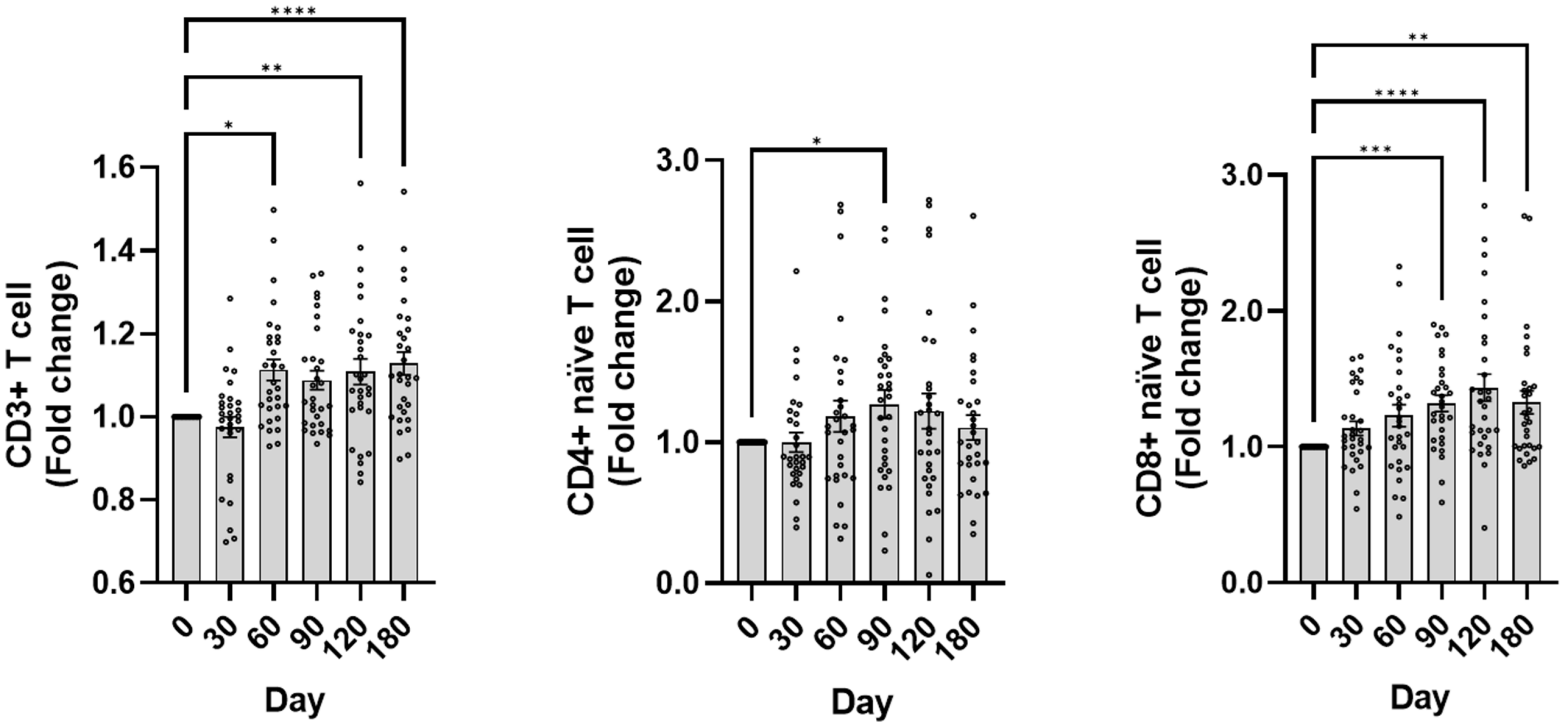
Changes in CD3+ T cells, naïve T cells following GHRH therapy. The graph depicts the fold change over time of CD3+ T cells, naïve CD4+ T cells (CD45RA+ CD62L+ CD4+), and naïve CD8+ T cells (CD45RA+ CD62L+ CD8+). The day 0 values were analyzed from blood samples collected before GHRH administration, and the GHRH administration was performed on day 0. Quantitative data are presented as mean ± SEM. Normality was tested using the Shapiro–Wilk test, and the Friedman test with Dunn’s multiple comparison test was applied for post hoc comparisons with day 0. Significance markers *, **, ***, and **** correspond to *p* < 0.05, *p* < 0.01, *p* < 0.001, and *p* < 0.0001 compared to day 0, respectively. The images shown were generated using GraphPad Prism version 10.

The impact of canine GHRH therapy on immune cells was also analyzed. CD3+ T-cell percentages in leukocytes were recorded at days 0, 30, 60, 90, 120, and 180, with respective mean ± SEM values of 83.12 ± 1.82, 81.31 ± 2.78, 91.31 ± 0.84, 89.44 ± 1.14, 90.69 ± 1.08, and 92.45 ± 0.87. To evaluate the variation in CD3+ T cells from day 0, fold change analysis was utilized, with findings depicted in Figure 5. Administration of a plasmid encoding GHRH resulted in a statistically significant change in the CD3^+^ T cell population over time (Friedman test, *p* < 0.0001), with *post hoc* analysis revealing significantly higher values at days 60, 120, and 180 compared with baseline (*p* < 0.05 for each).

Naïve CD4+ T-cell percentages in leukocytes were quantified at day 0, 30, 60, 90, 120, and 180, yielding means ± SEM of 33.33 ± 2.36, 31.53 ± 2.33, 35.87 ± 2.52, 38.70 ± 2.76, 36.85 ± 3.13, and 34.01 ± 2.35, respectively. A Friedman test revealed a significant change over time (*p* = 0.0255), with post hoc analysis (uncorrected Dunn’s test) showing a significant increase at day 90 compared with baseline (*p* < 0.05).

The proportion of naïve CD8+ T cells within leukocytes was evaluated across the same period, yielding percentages of 45.96 ± 3.98, 49.30 ± 3.93, 50.94 ± 3.72, 56.10 ± 3.91, 57.02 ± 3.59, and 54.86 ± 3.80 (mean ± SEM). A Friedman test revealed a significant change over time (*p* < 0.0001), and post hoc analysis (Dunn’s multiple comparison test) showed significant increases from day 90 through day 180 compared with baseline (all *p* < 0.005).

Additionally, Naïve CD8+ T-cell counts showed an upward trend by day 120 in both the IGF-1 Low Basal Group and High Basal Group (Supplementary Figure 2).

### 3.6 Safety assessment

Safety evaluations involved monitoring skin reactions at the drug application site, analyzing CBC and serum chemistry for abnormalities, verifying UPC, and assessing adverse event reports from owners during the 180-day clinical trial period. No dogs exhibited abnormal skin symptoms at the site of drug administration. One subject showed mild hind limb tremors following GHRH plasmid injection, which resolved without intervention.

Over the 180-day observation period, five treated dogs exhibited atypical CBC results. Dog No.14 showed mild anemia with a hematocrit (HCT) of 36.6% (reference range [RR]: 37.3–61.7%) on day 30; values at all other time points were within the RR. On day 120, two dogs (No.22 and No.29) exhibited mild thrombocytopenia with platelet counts of 126 K/μl and 120 K/μl, respectively (RR: 148–484 K/μl). Dog No.30 exhibited severe thrombocytopenia with a platelet count of 22 K/μl (RR: 148–484 K/μl) but no clinical symptoms. One dog (No.25) showed pancytopenia (HCT 33%, RR: 37.3–61.7%; PLT 35 K/μl, RR: 148–484 K/μl; WBC 3.95 K/μl, RR: 5.05–16.76 K/μl) without clinical symptoms. In all five dogs, hematologic values returned to within the RR at subsequent evaluations without any treatment.

During each evaluation, serum chemistry parameters were assessed, including total protein, albumin, globulin, ALT, AST, ALP, GGT, total bilirubin, amylase, lipase, glucose, total cholesterol, triglyceride, BUN, creatinine, calcium, phosphorus, Na+, K+, Cl−, and venous pH. Results indicated that ALP levels increased in two dogs, while one dog demonstrated elevated ALT levels. No further abnormalities were observed beyond these instances. In one case of elevated ALP, levels doubled the upper normal limit between day 30 and 180. In another case, ALP levels tripled the upper threshold between day 60 and day 120. In both cases, ALP values returned to within the reference range by day 180 without any treatment. For the dog with elevated ALT, levels rose to double the upper limit between day 90 and day 180. No abnormalities were found in the UPC test performed on the 30 senior dogs participating in the study on day 180.

Review of adverse event forms revealed that 11 dogs (36.7%) experienced vomiting and 9 dogs (30.0%) experienced diarrhea during the 180-day period. Across the study, the 9 dogs with diarrhea had an average of 4.8 episodes each, and the 11 dogs with vomiting had an average of 2.5 episodes each. Two dogs exhibited transient anorexia accompanied by diarrhea on the same day, and one dog showed transient anorexia with reduced vigor.

## 4 Discussion

Companion animals have become very important for our society, and studies have demonstrated significant benefits to humans (29, 30). There are estimated 700 million dogs worldwide (31). As in humans, any therapy that can improve the overall health of aging companion animals would be a significant advancement for veterinary medicine, with direct implications for human conditions. In this trial, we examined the therapeutic effectiveness and safety of administering a canine GHRH-encoding plasmid in 30 healthy senior dogs and followed them for 180 days. A key finding was the significant improvement in clinical scores, which encompassed various aspects of the dogs’ overall well-being and quality of life. Using the Friedman test with Dunn’s multiple comparison test, we found that clinical scores were significantly higher than baseline from mid-study through day 180. Owner-reported improvements in appetite, activity, and exercise tolerance showed a similar pattern. Overall, 90% of treated dogs showed improvement in clinical score. The findings are consistent with earlier research showing enhanced quality of life scores post GHRH plasmid therapy via electroporation in dogs and cats with chronic kidney disease (26), as well as improved psychological well-being, energy, and emotional response in adult humans with GH deficiency receiving GH replacement (32). Notably, the canine GHRH protein utilized in this study shows a high degree of sequence similarity to its human counterpart (Figure 1), suggesting the potential for comparable biological effects in humans. Given the observed improvements in clinical scores, minimal side effects, and bidirectional modulation of IGF-1 toward physiological ranges, it would be worthwhile to explore similar GHRH-encoding plasmid therapy in human clinical trials. Such an approach could open new avenues for mitigating immunosenescence and addressing age-related declines in health and immune function.

Regarding limb thickness, used here as a surrogate indicator of muscle strength, significant increases were observed only in the hindlimbs at day 180 (left hindlimb, *p* = 0.0168; right hindlimb, *p* = 0.0209 vs. baseline), while no significant changes were detected in either forelimb at any time point. This finding may indicate muscle mass augmentation in the hindlimbs over time, potentially contributing to improved physical function and aligning with the observed clinical score improvements. The observed pattern is consistent with results from human studies, which show that recombinant human growth hormone therapy increases muscle mass and decreases fat mass in GH-deficient patients, healthy elderly men, and malnourished older individuals (32–35). Animal studies have shown that delivering GHRH plasmid therapy via electroporation enhances body condition scores in pregnant Holstein heifers (16), as well as promoting weight gain while reducing fat accumulation and boosting bone mineral density in pigs (36). An increase in limb thickness may indicate muscle mass augmentation, potentially boosting physical well-being and life quality, and is congruent to the clinical score improvements.

Notably, when dogs were categorized into two groups post-hoc using a baseline IGF-1 threshold of 90 ng/mL, those with baseline IGF-1 levels below 90 ng/mL demonstrated a significantly greater rise in IGF-1 after GHRH administration (84.6% increase vs. 23.5%, *p* = 0.001). This finding aligns with a previous study in dogs with chronic kidney disease, where 72% showed an IGF-1 increase following GHRH plasmid administration via electroporation (26). Together, these results suggest that dogs with low baseline IGF-1 levels may be more responsive to GHRH plasmid therapy, potentially due to underlying GHRH deficiency.

Mary et al.’s research reported that older humans can exhibit GHRH deficiency, which was revealed by observing the response of GH secretion to GHRH after administering varying amounts of a GHRH receptor antagonist to both young and elderly men (37). In the 1990s, research focused on increasing GH and IGF-1 in the elderly via GHRH analogs like GH secretagogues or GH-releasing peptide mimetics. The findings showed elevated GH and IGF-levels following GHRH-analog administration (38, 39). Our research suggests that the negative effects of reduced IGF-1 due to aging-related GHRH deficiency might be mitigated by administering a plasmid that encodes for exogenous GHRH, and these effects are greater in population with lower IGF-1 levels prior to treatment. Additionally, this raises the possibility that administering GHRH, rather than direct IGF-1 supplementation, could allow for more physiologically regulated modulation of IGF-1 levels according to an individual’s baseline status, although further studies are required to confirm this.

Clinical research in human medicine indicates that recombinant human growth hormone (rhGH) administration to the elderly may positively affect body composition, such as muscle and fat mass, as well as bone mineral density and skin thickness (32–35). RhGH was thus regarded as an antiaging agent (40). Subsequent research has indicated a heightened risk of diabetes mellitus following GH therapy (41, 42). Although GH levels were not measured directly in this study, IGF-1 levels were monitored after administration of the GHRH-encoding plasmid and found that dogs with low initial IGF-1 levels increased and approached the average, while dogs with high initial levels tended to decrease and approach the average value. These findings imply that the administered GHRH plasmid does not act solely in a unidirectional manner to increase or decrease IGF-1 levels but rather serves as a bidirectional modulator—elevating IGF-1 when baseline levels are low, and reducing them when initially elevated. This regulatory behavior suggests that the GHRH-GH-IGF-1 axis can maintain physiological homeostasis through feedback-sensitive modulation. In addition, when comparing IGF-1 levels according to body weight, small dogs had average IGF-1 levels, but medium to large dogs had higher baseline IGF-1 levels but decreased closer to the average level (Supplementary Figure 1). This means that GHRH plasmid therapy does not cause persistent elevations of GH and IGF-1, but rather maintains optimal concentrations to promote homeostasis in the body. Similarly, a study administering plasmid-based GHRH therapy with electroporation to healthy young dogs showed that treated dogs gained more weight compared to controls, without altering blood glucose, insulin, or adrenocorticotropic hormone levels (25). Although GH was not directly measured in this study, our findings, in combination with previous research, did not reveal clear evidence of significant side effects related to increased GH/IGF-1 levels. Nevertheless, further research is warranted to comprehensively assess potential adverse effects and to elucidate the mechanism by which GHRH plasmid therapy may help maintain IGF-1 homeostasis in the body.

The flow cytometry results (Figure 4) corroborate the capability of GHRH plasmid therapy to counteract immunosenescence by modifying T-cell populations. The rise in CD3+ T cells, particularly in naïve CD4+ and CD8+ subsets, suggests a beneficial effect on immune health, possibly postponing the onset of immunosenescence. The study on GHRH supplementation through plasmid and electroporation in pregnant Holstein heifers showed an increase in CD2+ αβ T cells, CD23+ CD4+ cells, naïve T cells, and natural killer lymphocytes posttherapy (16) akin to the findings of the current research. Recombinant human GH therapy has been demonstrated to rejuvenate the thymus and boost naïve CD4+ and CD8+ T-cell counts in individuals with human immunodeficiency virus-1 (HIV-1) (12). Enhancing these T-cell subsets could bolster immune surveillance and pathogen response, crucial for protection from infections later in life.

Interestingly, the changes in IGF-1 by GHRH plasmid injection were modulate with an increase or decrease according to basal level, but the naïve CD8 T-cell count was analyzed as an increasing trend, confirming the double efficacy of the drug as a modulator to optimize the concentration of IGF-1 while increasing immunity. This is not a one-way treatment that only stimulates or inhibits as seen in existing drugs, but it can be controlled on an individual basis by using the homeostasis of GHRH-GH-IGF-1 axis.

The safety profile of GHRH plasmid therapy is generally favorable, though transient hematological abnormalities were noted in five cases, with two experiencing severe transient thrombocytopenia. This is similar to the results of other studies that have used GHRH plasmid injection in dogs (25, 26). This evidence highlights the importance of diligent surveillance and assessment in using this treatment method. Occasional instances of increased liver enzymes occurred, yet lacked consistent significance and a direct link to GHRH plasmid therapy remains unconfirmed. Some dogs experienced mild and transient gastrointestinal symptoms, including diarrhea and vomiting, during the study. However, digestive symptoms such as vomiting or diarrhea are nonspecific clinical manifestations that can occur due to dietary changes or stress in similarly aged animals. These symptoms did not occur immediately after the injection (the first 2 weeks after injection), but rather sporadically and transiently over the course of 180 days. Therefore, it is difficult to attribute these symptoms directly to the side effects of the GHRH encoding plasmid injection. The lack of adverse effects at the injection site is consistent with earlier research on GHRH therapy administered through plasmid and electroporation in dogs, pigs, and heifers. (16, 25, 36) In addition, concerns regarding potential tumor promotion were not supported by previous studies: GHRH plasmid injection did not increase tumor growth in mice with LL-2 lung adenocarcinoma or in nude mice with carcinomatosis (43, 44), and similarly, no tumor growth promotion was observed in elderly dogs with naturally occurring tumors following GHRH plasmid administration (45). The results highlight the need for ongoing studies on the enduring impacts and possible advantages of this novel treatment, as well as further research into potential adverse effects, including those related to repeated administration or individual-specific responses. Maintaining a balance between safety and efficacy is crucial for supporting healthy aging in dogs and the elderly.

One of the limitations of this study is that it was not a randomized, placebo-controlled trial. This design limitation could potentially introduce bias in the clinical scoring of the dogs, as the lack of a placebo group does not control for the placebo effect or observer bias. As a result, improvements in clinical scores might be overestimated, affecting the interpretation of the treatment’s efficacy. To mitigate this, we employed the Friedman test with Dunn’s multiple comparison test to analyze repeated measures within individuals over time. Another limitation is the underrepresentation of large breed dogs, with only two weighing over 25 kg and one over 40 kg. This is a significant limitation given that large breeds generally have different basal levels of IGF-1 compared to smaller breeds (46). The underrepresentation of large breed dogs in our sample could skew the findings and may not accurately reflect the effects of the treatment across all body sizes. Large breeds are particularly important to study due to their distinct physiological profiles and potential differences in treatment responses. Therefore, further research is needed to assess the effects of GHRH plasmid therapy in a more representative sample of large breed dogs, which could help generalize the findings to a broader canine population.

## 5 Conclusion

Our study demonstrates the potential of GHRH encoding plasmid therapy in improving overall well-being and immunosenescence-related changes in aging dogs. This intervention was associated with significant increases in the clinical well-being scores, potential limb muscle mass enhancement, and an increase in T cell populations. The study indicates that plasmid therapy encoding for GHRH could be a novel strategy for supporting healthy aging in canines, potentially extending lifespan and improving life quality in the aged dogs. Further research is necessary to understand the extended impacts, ensure long-term safety, and explore wider applications of this treatment method.

## Data Availability

The authors declare that all the data supporting the findings of this study are available within this article, its supplementary information files, or are available from the corresponding author, who has all relevant data.

## References

[1] Licastro F Candore G Lio D Porcellini E Colonna-Romano G Franceschi C. Innate immunity and inflammation in ageing: a key for understanding age-related diseases. Immunity Ageing. (2005) 2:8. doi: 10.1186/1742-4933-2-8, PMID: 15904534 PMC1166571

[2] Day M. Ageing, immunosenescence and inflammageing in the dog and cat. J Comp Pathol. (2010) 142:S60–S69. doi: 10.1016/j.jcpa.2009.10.011 20005526

[3] Pereira M Valério-Bolas A Saraiva-Marques C Alexandre-Pires G Pereira da Fonseca I Santos-Gomes G. Development of dog immune system: from in uterus to elderly. Vet Sci. (2019) 6:83. doi: 10.3390/vetsci6040083, PMID: 31640234 PMC6958461

[4] Thomas R Wang W Su D-M. Contributions of age-related thymic involution to immunosenescence and inflammaging. Immunity Ageing. (2020) 17:1–17. doi: 10.1186/s12979-020-0173-8, PMID: 31988649 PMC6971920

[5] Gruver A Hudson L Sempowski G. Immunosenescence of ageing. J Pathol. (2007) 211:144–56. doi: 10.1002/path.2104, PMID: 17200946 PMC1931833

[6] Aw D Silva AB Palmer DB. Immunosenescence: emerging challenges for an ageing population. Immunology. (2007) 120:435–46. doi: 10.1111/j.1365-2567.2007.02555.x, PMID: 17313487 PMC2265901

[7] Withers SS Moore PF Chang H Choi JW McSorley SJ Kent MS. Multi-color flow cytometry for evaluating age-related changes in memory lymphocyte subsets in dogs. Dev Comp Immunol. (2018) 87:64–74. doi: 10.1016/j.dci.2018.05.022, PMID: 29859828 PMC6197816

[8] Watabe A Fukumoto S Komatsu T Endo Y Kadosawa T. Alterations of lymphocyte subpopulations in healthy dogs with aging and in dogs with cancer. Vet Immunol Immunopathol. (2011) 142:189–200. doi: 10.1016/j.vetimm.2011.05.008, PMID: 21680028

[9] Reis AB Carneiro CM das Gracas Carvalho M Teixeira-Carvalho A Giunchetti RC Mayrink W. Establishment of a microplate assay for flow cytometric assessment and it is use for the evaluation of age-related phenotypic changes in canine whole blood leukocytes. Vet Immunol Immunopathol. (2005) 103:173–85. doi: 10.1016/j.vetimm.2004.08.014, PMID: 15621304

[10] Bodart G Farhat K Charlet-Renard C Salvatori R Geenen V Martens H. The somatotrope growth hormone-releasing hormone/growth hormone/insulin-like growth factor-1 axis in immunoregulation and immunosenescence. Endocr Immunol. (2017) 48:147–59. doi: 10.1159/000452913 28245459

[11] Burgess W Liu Q Zhou J-H Tang Q Ozawa A VanHoy R. The immune-endocrine loop during aging: role of growth hormone and insulin-like growth factor-I. Neuroimmunomodulation. (1999) 6:56–68. doi: 10.1159/000026365, PMID: 9876236

[12] José EB Miguel G-M. Growth hormone, immunosenescence and vaccination failure in the elderly. Clin Immunol Commun. (2023) 3:51–7. doi: 10.1016/j.clicom.2023.02.005

[13] Savino W Mendes-da-Cruz DA Lepletier A Dardenne M. Hormonal control of T-cell development in health and disease. Nat Rev Endocrinol. (2016) 12:77–89. doi: 10.1038/nrendo.2015.168, PMID: 26437623

[14] Khorram O Yeung M Vu L Yen SSC. Effects of [Norleucine27]growth hormone-releasing hormone (GHRH) (1–29)-NH2 administration on the immune system of aging men and women1. J Clin Endocrinol Metab. (1997) 82:3590–6. doi: 10.1210/jcem.82.11.4363, PMID: 9360512

[15] Murphy WJ Durum SK Longo D. Role of neuroendocrine hormones in murine T cell development. Growth hormone exerts thymopoietic effects in vivo. J Immunol. (1992) 149:3851–7. doi: 10.4049/jimmunol.149.12.3851, PMID: 1460277

[16] Brown PA Davis WC Draghia-Akli R. Immune-enhancing effects of growth hormone-releasing hormone delivered by plasmid injection and electroporation. Mol Ther. (2004) 10:644–51. doi: 10.1016/j.ymthe.2004.06.1015, PMID: 15451448

[17] Koo GC Huang C Camacho R Trainor C Blake JT Sirotina-Meisher A. Immune enhancing effect of a growth hormone secretagogue. J Immunol. (2001) 166:4195–201. doi: 10.4049/jimmunol.166.6.4195, PMID: 11238671

[18] Taub DD Longo DL. Insights into thymic aging and regeneration. Immunol Rev. (2005) 205:72–93. doi: 10.1111/j.0105-2896.2005.00275.x, PMID: 15882346

[19] Bellows J Colitz CMH Daristotle L Ingram DK Lepine A Marks SL. Common physical and functional changes associated with aging in dogs. J Am Vet Med Assoc. (2015) 246:67–75. doi: 10.2460/javma.246.1.67, PMID: 25517328

[20] The Ohio State University Veterinary Medical Center Honoring the Bond Program. How will I know? Assessing quality of life and making difficult decisions for your pet. Columbus, OH: Ohio State University (2024).

[21] Villalobos AE. Quality-of-life assessment techniques for veterinarians. Clin Tech Small Anim Pract. (2005) 20:45–6. doi: 10.1016/j.cvsm.2011.03.013 21601744

[22] Guex N Peitsch MC Schwede T. Automated comparative protein structure modeling with SWISS-MODEL and Swiss-PdbViewer: a historical perspective. Electrophoresis. (2009) 30:S162–73. doi: 10.1002/elps.200900140, PMID: 19517507

[23] Cong Z Zhou F Zhang C Zou X Zhang H Wang Y. Constitutive signal bias mediated by the human GHRHR splice variant 1. Proceed National Acad Sci. (2021) 118:e2106606118. doi: 10.1073/pnas.2106606118, PMID: 34599099 PMC8501799

[24] Zhou F Zhang H Cong Z Zhao L-H Zhou Q Mao C. Structural basis for activation of the growth hormone-releasing hormone receptor. Nat Commun. (2020) 11:5205. doi: 10.1038/s41467-020-18945-0, PMID: 33060564 PMC7567103

[25] Draghia-Akli R Cummings K Khan A Brown P Carpenter R. Effects of plasmid-mediated growth hormone releasing hormone supplementation in young, healthy beagle dogs. J Anim Sci. (2003) 81:2301–10. doi: 10.2527/2003.8192301x, PMID: 12968706

[26] Brown PA Bodles-Brakhop AM Pope MA Draghia-Akli R. Gene therapy by electroporation for the treatment of chronic renal failure in companion animals. BMC Biotechnol. (2009) 9:1-13. doi: 10.1186/1472-6750-9-4, PMID: 19149896 PMC2663557

[27] Monjazeb AM Kent MS Grossenbacher SK Mall C Zamora AE Mirsoian A. Blocking indolamine-2, 3-dioxygenase rebound immune suppression boosts antitumor effects of radio-immunotherapy in murine models and spontaneous canine malignancies. Clin Cancer Res. (2016) 22:4328–40. doi: 10.1158/1078-0432.CCR-15-3026, PMID: 26979392 PMC5010514

[28] Bismarck D Schütze N Moore P Büttner M Alber G Buttlar H. Canine CD4+ CD8+ double positive T cells in peripheral blood have features of activated T cells. Vet Immunol Immunopathol. (2012) 149:157–66. doi: 10.1016/j.vetimm.2012.06.014, PMID: 22789871

[29] Barker SB Wolen AR. The benefits of human–companion animal interaction: a review. J Vet Med Educ. (2008) 35:487–95. doi: 10.3138/jvme.35.4.487, PMID: 19228898

[30] Friedmann E Son H. The human–companion animal bond: how humans benefit. Vet Clin North Am Small Anim Pract. (2009) 39:293–326. doi: 10.1016/j.cvsm.2008.10.015, PMID: 19185195

[31] Hughes J Macdonald DW. A review of the interactions between free-roaming domestic dogs and wildlife. Biol Conserv. (2013) 157:341–51. doi: 10.1016/j.biocon.2012.07.005

[32] Gibney J Wallace J Spinks T Schnorr L Ranicar A Cuneo R. The effects of 10 years of recombinant human growth hormone (GH) in adult GH-deficient patients. J Clin Endocrinol Metab. (1999) 84:2596–602. doi: 10.1210/jcem.84.8.5916, PMID: 10443645

[33] Taaffe DR Pruitt L Reim J Hintz RL Butterfield G Hoffman AR. Effect of recombinant human growth hormone on the muscle strength response to resistance exercise in elderly men. J Clin Endocrinol Metab. (1994) 79:1361–6. doi: 10.1210/jcem.79.5.7525633, PMID: 7525633

[34] Salomon F Cuneo RC Hesp R Sönksen PH. The effects of treatment with recombinant human growth hormone on body composition and metabolism in adults with growth hormone deficiency. New Engl J Med. (1989) 321:1797–803. doi: 10.1056/NEJM198912283212605, PMID: 2687691

[35] Kaiser FE Silver AJ Morley JE. The effect of recombinant human growth hormone on malnourished older individuals. J Am Geriatr Soc. (1991) 39:235–40. doi: 10.1111/j.1532-5415.1991.tb01643.x, PMID: 2005335

[36] Draghia-Akli R Ellis KM Hill LA Malone PB Fiorotto ML. High-efficiency growth hormone releasing hormone plasmid vector administration into skeletal muscle mediated by electroporation in pigs. FASEB J. (2003) 17:1–17. doi: 10.1096/fj.02-0671fje, PMID: 12514110

[37] Russell-Aulet M Jaffe CA DeMott-Friberg R Barkan AL. In vivo semiquantification of hypothalamic growth hormone-releasing hormone (GHRH) output in humans: evidence for relative GHRH deficiency in aging*. J Clin Endocrinol Metab. (1999) 84:3490–7. doi: 10.1210/jc.84.10.3490, PMID: 10522985

[38] Chapman IM Bach MA Van Cauter E Farmer M Krupa D Taylor AM. Stimulation of the growth hormone (GH)-insulin-like growth factor I axis by daily oral administration of a GH secretogogue (MK-677) in healthy elderly subjects. J Clin Endocrinol Metab. (1996) 81:4249–57. PMID: 8954023 10.1210/jcem.81.12.8954023

[39] Chapman IM Hartman ML Pezzoli SS Thorner MO. Enhancement of pulsatile growth hormone secretion by continuous infusion of a growth hormone-releasing peptide mimetic, L-692,429, in older adults--a clinical research center study. J Clin Endocrinol Metab. (1996) 81:2874–80. PMID: 8768844 10.1210/jcem.81.8.8768844

[40] Junnila RK List EO Berryman DE Murrey JW Kopchick JJ. The GH/IGF-1 axis in ageing and longevity. Nat Rev Endocrinol. (2013) 9:366–76. doi: 10.1038/nrendo.2013.67, PMID: 23591370 PMC4074016

[41] Schauster AC Geletko SM Mikolich DJ. Diabetes mellitus associated with recombinant human growth hormone for HIV wasting syndrome. Pharmacotherapy: J human Pharmacol. Drug Ther. (2000) 20:1129–34. doi: 10.1592/phco.20.13.1129.35037, PMID: 10999508

[42] Liu H Bravata DM Olkin I Nayak S Roberts B Garber AM. Systematic review: the safety and efficacy of growth hormone in the healthy elderly. Ann Intern Med. (2007) 146:104–15. doi: 10.7326/0003-4819-146-2-200701160-00005, PMID: 17227934

[43] Khan AS Anscombe IW Cummings KK Pope MA Smith LC Draghia-Akli R. Effects of plasmid-mediated growth hormone-releasing hormone supplementation on LL-2 adenocarcinoma in mice. Mol Ther. (2003) 8:459–66. doi: 10.1016/S1525-0016(03)00175-8, PMID: 12946319

[44] Khan AS Smith LC Anscombe IW Cummings KK Pope MA Draghia-Akli R. Growth hormone releasing hormone plasmid supplementation, a potential treatment for cancer cachexia, does not increase tumor growth in nude mice. Cancer Gene Ther. (2005) 12:54–60. doi: 10.1038/sj.cgt.7700767, PMID: 15375378

[45] Tone CM Cardoza DM Carpenter RH Draghia-Akli R. Long-term effects of plasmid-mediated growth hormone releasing hormone in dogs. Cancer Gene Ther. (2004) 11:389–96. doi: 10.1038/sj.cgt.7700717, PMID: 15073611

[46] Jaillardon L Martin L Nguyen P Siliart B. Serum insulin-like growth factor type 1 concentrations in healthy dogs and dogs with spontaneous primary hypothyroidism. Vet J. (2011) 190:e95–9. doi: 10.1016/j.tvjl.2011.03.020, PMID: 21546289

