# Supplementary material for: Wellness-enhancing effects of the canine growth hormone releasing hormone therapy mediated by plasmid and electroporation in healthy old dogs
Source: Front Vet Sci. 2025 Sep 15;12:1609405. doi: 10.3389/fvets.2025.1609405 (PMC12477696; doi:10.3389/fvets.2025.1609405)
Supplement: Supplementary file 1 [file Table_1.DOCX]

Supplementary Material


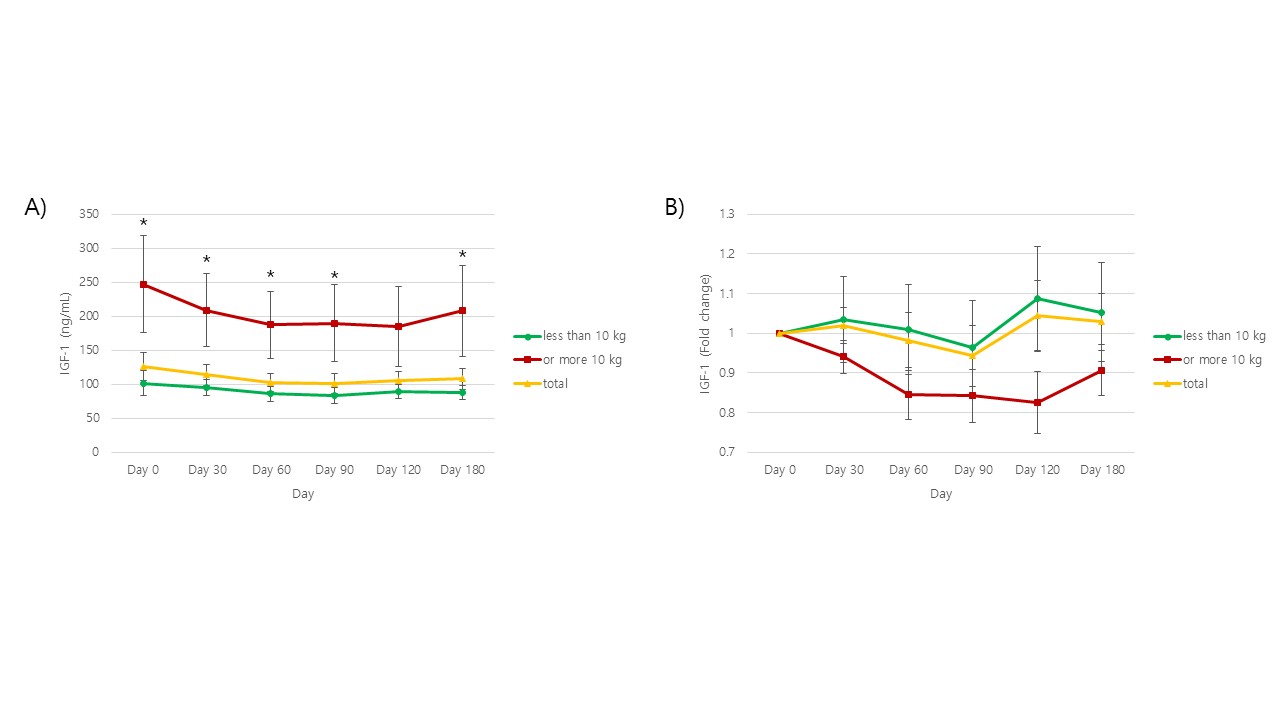


**Supplementary Figure 1.** Changes in IGF-1 levels following GHRH treatment based on body weight

A) Mean IGF-1 levels in total dogs (n=30), Small dogs (less than 10 kg) Group (n=25), and Medium to Large dogs (10 kg or more) Group (n=5) by time point. B) Mean changes in IGF-1 levels converted to fold change over time graph.

Graphs represent the mean ± SEM. The day 0 values were analyzed from blood samples collected before GHRH administration, and the GHRH administration was performed on day 0. * total Vs. p <0.05


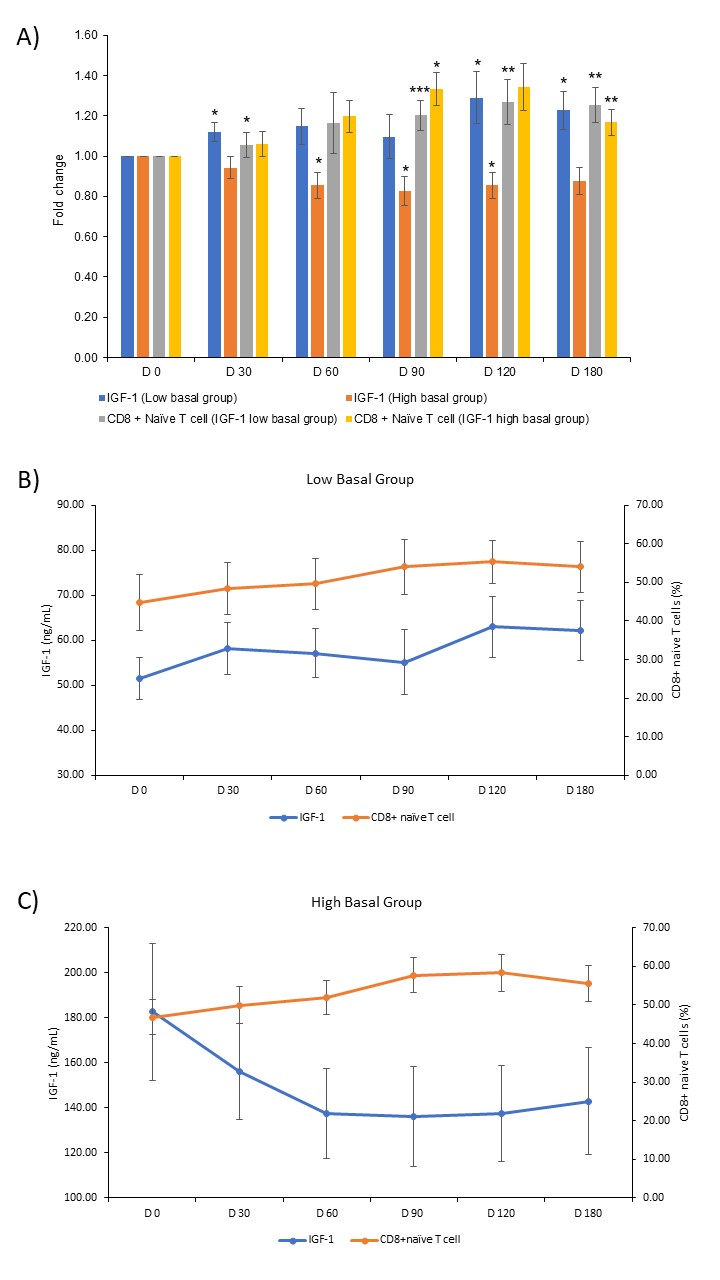


**Supplementary Figure 2.** Comparison of IGF-1 and CD8 naive T cells by classification based on canine standard IGF-1

A) Fold change graph by time point by classifying IGF-1 and CD8 naive T cells based on canine standard IGF-1.

B) Comparison of IGF-1 and CD8 naive T cells in the Low Basal Group (serum IGF-1<90 ng/mL).

C) Comparison of IGF-1 and CD8 naive T cells in the High Basal Group (serum IGF-1>90 ng/mL).

Graphs represent the mean ± SEM. The day 0 values were analyzed from blood samples collected before GHRH administration, and the GHRH administration was performed on day 0. Each Day 0 Vs. *p <0.05; **p <0.01; ***p <0.05
